# Integrated Patient Digital and Biomimetic Twins for Precision Medicine: A Perspective

**DOI:** 10.1055/a-2649-1560

**Published:** 2025-07-23

**Authors:** Mark T. Miedel, Mark E. Schurdak, Andrew M. Stern, Alejandro Soto-Gutierrez, Eric von Strobl, Jaideep Behari, D. Lansing Taylor

**Affiliations:** 1 Department of Pharmacology and Chemical Biology, Organ Pathobiology and Therapeutics Institute, University of Pittsburgh, Pittsburgh, Pennsylvania; 2 Department of Computational and Systems Biology, Organ Pathobiology and Therapeutics Institute, University of Pittsburgh, Pittsburgh, Pennsylvania; 3 Department of Pathology, Center for Transcriptional Medicine, University of Pittsburgh, Pittsburgh, Pennsylvania; 4 Department of Biomedical Informatics, University of Pittsburgh, Pittsburgh, Pennsylvania; 5 Division of Gastroenterology, Hepatology and Nutrition, Department of Medicine, University of Pittsburgh, Pittsburgh, Pennsylvania

**Keywords:** patient digital twins, patient biomimetic twins, precision medicine, organoids, induced pluripotent stem cells, microphysiological systems, pathophysiology of complex diseases, biomarker discovery

## Abstract

A new paradigm for drug development and patient therapeutic strategies is required, especially for complex, heterogeneous diseases, including metabolic dysfunction-associated steatotic liver disease (MASLD). Heterogeneity in MASLD patients is driven by genetics, various comorbidities, gut microbiota composition, lifestyle, environment, and demographics that produce multiple patient disease presentations and outcomes. Existing drug development methods have had limited success for complex, heterogeneous diseases like MASLD where only a fraction of patients respond to specific treatments, prediction of a therapeutic response is not presently possible, and the cost of the new classes of drugs is high. However, it is now possible to generate patient digital twins (PDTs) that are computational models of patients using clinomics and other “omics” data collected from patients to make various predictions, including responses to therapeutics. PDTs are then integrated with patient biomimetic twins (PBTs) that are patient-derived organoids or induced pluripotent stem cells that are then differentiated into the optimal number of organ-specific cells to produce organ experimental models. The PBTs mimic key aspects of the patient's pathophysiology, enabling predictions to be tested. In conclusion, integration of PTDs and PBTs has the potential to create a powerful precision medicine platform, yet there are challenges.

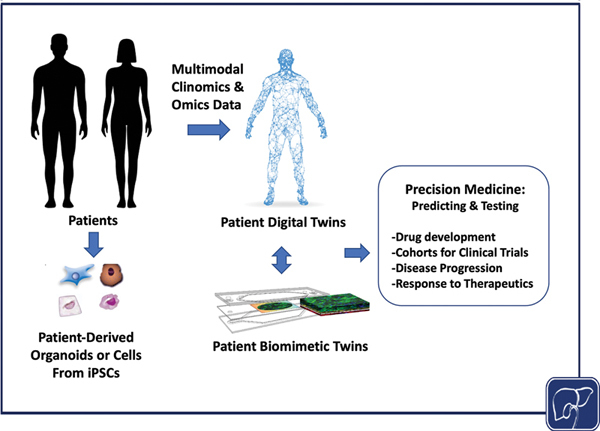

This perspective explores the opportunities and challenges in developing a precision medicine platform that addresses patient heterogeneity, a major challenge for drug discovery, development (including clinical trials), and patient care. The recent U.S. Food and Drug Administration (FDA) Modernization Act 2.0 facilitates the development of a potentially transformative precision medicine platform that integrates two “NAMs” that can be part of regulatory submissions to the FDA. 1 2 3 PDTs are computational models using multiomic data collected from the same individual patients over time 4 5 6 7 8 9 10 and PBTs are microphysiological systems (MPS) using patient-derived cells from the same patient's used to create the PDTs ( Fig. 1 ). 11 The combined and integrated PDTs and PBTs are harnessed to iteratively make and to test predictions about a patient such as probability of efficacy and safety of a drug, risk stratification, probability of success if enrolled in a given clinical trial and the selection of an optimal patient therapeutic strategy before making recommendations to the patient. While the integration of PDTs and PBTs holds great potential, some challenges remain.

**Fig. 1:**
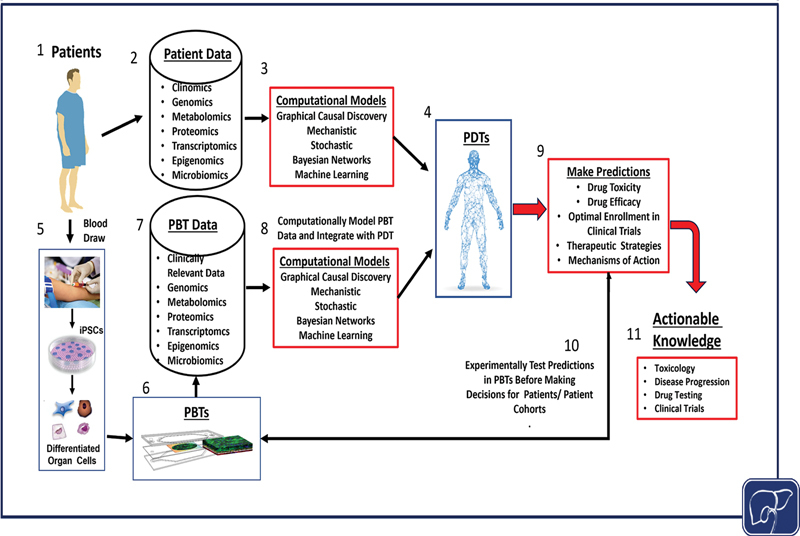
Integration of patient digital twins and patient biomimetic twins: data, modeling, and experimental testing of predictions increases the power of precision medicine. (1) Patients enrolled in a clinic have (2) optimal amount of data collected and saved in a database, and (3) computational models are created using a range of computational methods applied to multimodal datasets. (4) Patient digital twins (PDTs) are generated with the data and model continually updated during the course of care. (5) Patient blood is drawn and used to collect peripheral blood mononuclear cells (PBMCs) to generate induced pluripotent stem cells (iPSCs) from which differentiated organ cells are produced and/or organoids are created from patient tissue samples or iPSCs to create (6) patient biomimetic twins (PBTs) that mimic selected functions and characteristics of the patient. (7) Optimal amount of data is collected and saved from the PBTs in a database (distinct or the same as the PDT database) and (8) complementary computational models are created using the same/similar computational tools as applied to the PDTs. The PDTs and PBTs models are integrated and (9) predictions are made. (10) There is an iterative loop underlying the integration of PDTs and PBTs that is the key to the platform. Predictions are tested in the PBTs before finalizing the predictions to apply to the patients/patient cohorts as (11) actionable knowledge/Precision Medicine Platform.

Heterogeneity in complex, heterogeneous diseases such as metabolic dysfunction-associated steatotic liver disease (MASLD) is driven by genetics, various comorbidities, lifestyle, environment, demographics, and gut microbiota composition that produce multiple patient disease presentations and outcomes. MASLD is therefore an ideal clinical model for developing and implementing the integrated PDT and PBT precision medicine platform to get the right therapy to the right patient at the right time. Comorbidities are a particular challenge for MASLD patients: (1) the liver plays a key role in other metabolic disorders like type 2 diabetes (T2D), chronic kidney disease (CKD), and cardiovascular disease (CVD), 12 13 14 15 16 17 18 with MASLD preceding extrahepatic dysfunction in most cases. 19 20 21 (2) MASLD affects approximately 30% of the global populations, with 8 to 10% developing metabolic dysfunction-associated steatohepatitis (MASH), increasing risks of cirrhosis, hepatic decompensation, worsening of any comorbidity and hepatocellular carcinoma. 22 (3) MASLD is closely linked to other chronic metabolic disorders and can be a comorbidity with obesity, T2D, CVD, and CKD, all of which share common lifestyle, environmental, and pathophysiological risk factors (such as insulin resistance and systemic inflammation) and constitute the recently named cardiovascular-kidney-metabolic (CKM) syndrome. However, the CKM syndrome is better conceptualized as the cardiovascular-renal-hepatic-metabolic syndrome, given the critical importance of the liver in metabolic homeostasis ( Fig. 2 ). 23 24 25 Notably, 70% of patients with T2D and 90% with severe obesity also have MASLD. 26 27 28 Conversely, development of MASLD increases the risk of incident T2D. 29 (4) A robust worldwide MASLD/MASH drug development pipeline includes over 500 clinical trials. 30 31 32 However, there is a great challenge to develop therapeutics that will successfully treat the heterogeneous MASLD patient population.

**Fig. 2:**
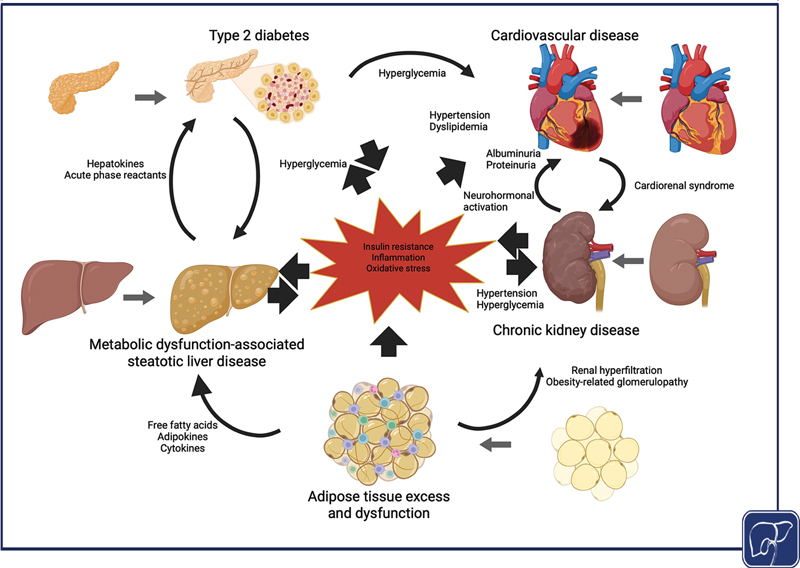
Conceptual model of the cardiovascular–kidney–metabolic syndrome. Chronic metabolic conditions such as cardiovascular disease (CVD), chronic kidney disease (CKD), and type 2 diabetes (T2D) commonly occur together. The term cardiovascular–kidney–metabolic (CKM) syndrome was proposed in 2023, recognizing the shared pathophysiological mechanisms among these disorders. The origin of CKM syndrome is proposed to be dysfunctional and/or excessive adipose tissue that promotes systemic inflammation, insulin resistance, and oxidative stress, leading to chronic kidney disease, T2D, and ultimately cardiovascular disease. However, the CKM syndrome is better conceptualized as the cardiovascular–renal–hepatic–metabolic syndrome, given the critical importance of the liver in metabolic homeostasis. Metabolic dysfunction-associated steatotic liver disease (MASLD), the excessive deposition of fat in the liver, demonstrates a bidirectional relationship with T2D, with risk of T2D increasing with increasing liver fibrosis, and conversely, risk of liver fibrosis increasing with development of T2D. MASLD is also associated with increased risk of incident CVD and CKD. This framework highlights the importance of integrated management strategies that target the underlying common pathophysiological processes rather than each disorder individually. Created in BioRender. Behari (2025) https://BioRender.com/gurjbq2 .

Resmetirom, a thyroid hormone receptor β (THRb) agonist was conditionally approved by the FDA in 2024 for treatment of MASLD/MASH with F2/F3 fibrosis. 33 This conditional approval came after the pivotal Phase 3 MAESTRO-NASH clinical trial, in which 933 patients were enrolled, the primary study endpoints of at least 1 stage fibrosis regression was achieved in 26% of patients on resmetirom compared with 14% on placebo, demonstrating the small percentage of patients that respond! 34 In addition, resmetirom costs tens of thousands of dollars annually, 35 and future drugs are also projected to be costly. Furthermore, due to patient heterogeneity, no single drug is expected to work for all patients, all disease stages, and could be associated with side effects, including serious cases of hepatotoxicity in some patients. Thus, stakeholders, including patients, clinicians, insurers, and pharmaceutical companies, need to determine those individual patients who would optimally benefit from a particular drug/combination.

## Major Challenges in Managing the Heterogeneity of Patients with Metabolic Dysfunction-Associated Steatotic Liver Disease

Managing the heterogeneity of MASLD patients in clinical practice presents multiple challenges that require a precision medicine solution. (1) Screening and early detection of at-risk patients: identifying high-risk patients (e.g., those with obesity and T2D) and detecting advanced fibrosis early is crucial for timely intervention. (2) Initial risk stratification and determination of disease stage: despite the association of severe obesity and T2D with MASLD, risk stratification and disease staging remain difficult due to limitations in diagnostic tools like elastography and serum biomarkers mechanistically linked to the pathophysiology, especially in high-body mass index (BMI) individuals. (3) Prediction of risk and rate of liver disease progression: MASLD patients may be diagnosed at early, mid, or late disease stages. Since age (a surrogate for duration of the disease) is strongly associated with advanced disease, diagnosis of MASLD at an early stage poses the clinical challenge of predicting future risk of disease progression, as well as determining the rate of disease progression. Currently, predicting progression rate and risk remains difficult, with no effective stratification for rapid versus slow progressors to tailor surveillance based on risk profile. (4) Prediction of the development of extrahepatic complications associated with MASLD. While MASLD is closely linked to extrahepatic complications such as T2D, CKD, and CVD with advancing liver fibrosis, predicting which patients will develop these extrahepatic organ dysfunction remains uncertain. (5) Prediction of response to drug therapy/combinations: The MASLD/MASH drug development pipeline is robust with multiple therapies and combination therapies in advanced clinical trials. However, prediction of response to treatment is also complex, as most emerging therapies, including resmetirom (ca. 26% response rate) 34 and semaglutide (ca. 37% response rate), 36 benefit only subsets of patients. (6) Prediction of liver toxicity associated with MASH therapy. New therapies, including resmetirom, carry potential hepatotoxicity risks, necessitating a precision medicine platform to prevent drug-induced liver injury.

Due to the present lack of good predictive tools, current guidelines recommend initiating treatment with resmetirom in patients with MASLD/MASH and F2/F3 fibrosis. It is further recommended to monitor after 3 months for drug-induced liver injury, and noninvasive liver disease assessment at 1 year to monitor for treatment response based on improvement in noninvasive tests (NITs) of liver fibrosis, nonresponse to treatment based on worsening of NITs, or stability of disease course (no change in NITs). 37 However, in the absence of a precision medicine platform to identify patients with a high probability of a safe response only a small percentage of patients will be successfully treated and at a high cost and risk. Several other therapies targeting pathways in the liver are in development, including agonists for FGF21, FXR, PPAR pathways, as well as components of the inflammatory and fibrogenesis pathways. 31 In addition, Nutrient Stimulated Hormone-based therapies, including the GLP-1 receptor agonist semaglutide, GLP-1/GIP dual agonist, tirzepatide, GLP1RA/GIP/GRA triple agonist, retatrutide, are showing some efficacy for MASLD/MASH and are in advanced stages of development. Availability of these drugs with effects on multiple organ systems represent an exciting convergence of therapies for MASLD/MASH, obesity, T2D, CVD, and CKD. However, this further highlights the need for a precision medicine platform for predicting and testing for efficient and successful patient outcomes.

## Background to the Development of Patient Digital and Biomimetic Twins

The history of developing and applying integrated computational and experimental models has roots in the application of quantitative systems pharmacology (QSP) to drug discovery and development. 38 39 40 An important step toward the integration of computational and experimental models was integrating the principle of QSP with human MPS constructed with normal or diseased cells. 11 41 42 Interestingly, digital twin technology was initially used in industries like aerospace to create digital representations of physical entities such as rockets and planes to predict physical responses before testing the predictions “experimentally.” 43 By analogy, the large amount of data that can now be collected from patients has made it possible to create “PDTs.” A PDT may be defined as “a viewable digital replica of a patient, organ, or biological system that contains multidimensional, patient-specific information, and informs decisions.” 44 PDTs have been applied to patient data from multiple diseases for several years to make predictions about toxic liabilities and disease progression. 6 7 10 44 45 46

**Fig. 3:**
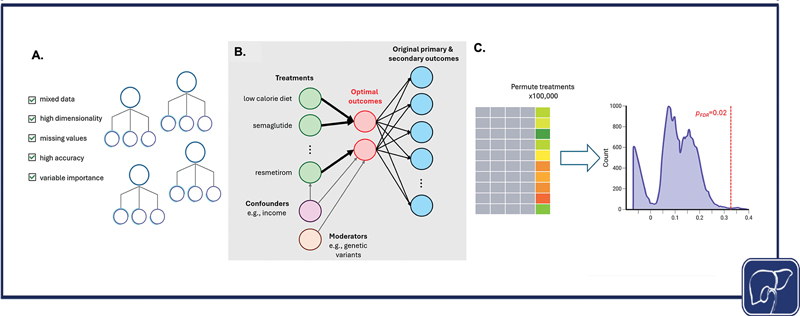
Computational approaches for patient digital twins (PDTs). ( **A** ) Random Forest and XGBoost tree models satisfy all necessary criteria needed to accurately model tabular biomedical data while remaining interpretable. ( **B** ) The Supervised Varimax (SV) algorithm compresses multiple outcome measures (blue) into a small set of optimal outcomes (red) that maximally differentiate between treatments (green). The effect size from treatments to optimal outcomes is large (bolded arrows) and has minimal overlap. Covariate adjustment will account for confounders to isolate causal effects of treatment from observational data. Covariate adjustment will also account for moderators that modify the true causal effects of treatment. The resultant model yields PDTs, where we can experiment on the moderators in silico and then test the findings in vitro using the patient-specific patient biomimetic twins (PBTs). The algorithm accommodates confounders and moderators to isolate the causal effects of treatments personalized toward each patient. ( **C** ) Permutation testing enables accurate assessment of the statistical significance of moderator effects by estimating *p* -values with correction for multiple comparisons.

The development of computational approaches to tackle the complexity and variability of MASLD patients is a major challenge and a variety of methods have been applied. 47 48 49 Most early approaches have attempted to match each patient to the optimal therapeutic treatment by learning large prediction models that predict a single outcome measure, such as the degree of liver fibrosis, using different treatments. 47 48 49 However, single outcome measures unfortunately fail to comprehensively capture the complexity of MASLD patients. Optimally, multiple outcome measures are applied to learn multivariate response profiles that differentiate treatments with large effect sizes from observational data. A comprehensive set of primary and secondary outcome measures are collected and are compressed into a few optimal outcomes that maximally differentiate between treatments (see Fig. 3 below). Compression is achieved by performing principal component analysis on the outcome measures to create outcomes that explain the most variance of the original primary and secondary outcomes 50 Optimal outcomes refer to the compressed and optimally rotated primary and secondary outcomes. An integrated statistical technique called Supervised Varimax (SV) that learns such optimal outcomes has been developed and applied. 51 52 53 Moderators and confounders are adjusted by incorporating them as covariates into the SV model. This facilitates the development of causal PDTs for making predictions, with results tested using the PBTs.

Experimental models have evolved from the use of animals, simple 2D and 3D cell cultures to multicell, 3D human MPS. MPS are “miniaturized functional units of organs with multiple cell types from animals or humans under a variety of physical and biochemical cues that recapitulate at least some organ and organ system physiology/pathophysiology.” 11 41 Single organ and coupled, multiorgan MPS have been constructed with primary cells, organoids, and induced pluripotent stem cells (iPSC)-derived patient cells for both high throughput and high content applications using microplates and microfluidic chips. 11 41 54 55 56 57 58 59 60 61 High content, structured, biomimetic human liver MPS including both the liver acinus microphysiological system (LAMPS) and the vascularized liver acinus microphysiological system (vLAMPS) are well published and validated platforms for investigating human liver biology, toxicology, and disease. 11 41 42 62 63 64 65 66 67 68 69 Both platforms presently use four human liver cell types (hepatocytes, liver sinusoidal endothelial cells (LSECS), hepatic stellate cells (HSCs) and Kupffer cells–macrophages). MPS constructed with patient-specific cells generated from iPSCs are called PBTs. The structured biomimetic human liver PBTs are compatible with a large set of phenotypic and molecular level live cell, secretome, and fixed endpoint readouts ( Table 1 ) that can be used to correlate with the collected patient data metrics and multiomics ( Table 2 ). 11 41

**Table 1:** Phenotypic and functional metrics to characterize metabolic dysfunction-associated steatotic liver disease progression and drug response in patient biomimetic twins

Readout	Measurement	MASLD relevance
Albumin secretion	ELISA	Metabolic competence, overall hepatocyte function
Urea secretion	Colorimetric	Metabolic competence, overall hepatocyte function
LDH release	Colorimetric	Hepatocellular injury corresponding with ALT and AST
Hepatocellular steatosis	LipidTOX staining	Normal and/or pathological fat storage in hepatocytes that correlates with clinical steatosis
Pro-inflammatory cytokine release	Multiplexed ELISA for TNFα, IL-6 and IL-1β	Kupffer cell activation; inflammatory response that correlates with serum levels
Stellate cell activation/fibrosis	α-SMA immunofluorescence	Fibrosis indicator that correlates with clinical liver fibrosis measurements
Pro-Collagen 1A1 secretion	ELISA	
Metabolomics/Lipidomics	GC/MS, LC/MS, (RP and HILIC)	Cellular and lipid metabolism profiles that correlate with clinical profiles associated with disease state and drug response

Abbreviations: ALT, alanine aminotransferase; AST, aspartate aminotransferase; ELISA, enzyme-linked immunosorbent assay; GC/MS, Gas chromatography–mass spectrometry; IL-1β, Interleukin-1 β; IL-6, Interleukin-6; LC/MS, liquid chromatography–mass spectrometry; MASLD, metabolic dysfunction-associated steatotic liver disease; RP/HILIC, reversed-phase liquid chromatography/hydrophilic interaction chromatography; TNFα, tumor necrosis factor α; α-SMA, α smooth muscle actin.

**Table 2:** Patient clinomics data being collected

Clinical data domains	Measurement methods	Variables	Data location
Demographics	Electronic health records	Age, gender, race/ethnicity	EHR
Morphometrics	Clinical scale, measuring tape, EKG, Dynamometer,	Height, weight, waist circumference, BMI (calculated), waist/height ratio (WHR), bone density, muscle strength	REDCap
Medical history and comorbidities	Electronic health records	Medical: diabetes, hypertension, dyslipidemia, coronary artery disease, obesity, hypothyroidism, sleep apnea, polycystic ovary, hypogonadism, hypopituitarism, gout, cholelithiasis, cancer. Surgical: cholecystectomy, weight loss surgery, appendectomy, tonsillectomy, bowel resection	EHR
Body composition	Body impedance analyzer, DEXA scan, MRI scan	Height, weight, fat mass, lean mass, total body water. Automated imaged analysis for visceral and subcutaneous adipose tissue (MRI—see below)	EHR
Medications	Electronic health records	Medications for diabetes, hypertension, dyslipidemia, weight loss, NASH, heart disease, steroids, gout	EHR
Biochemistries	Electronic health records, per protocol study visits	Hemoglobin, platelet count, ALT, AST, Total bilirubin, albumin, alkaline phosphatase, GGT, PT/INR, total cholesterol, LDL-C, HDL-C, triglyceride, BUN/creatinine	EHR
Continuous glucose monitoring	Dexacom Stelo personal glucose biosensor	Continuous glucose monitoring record over 7 d (per year).	REDCap
Patient Reported Outcome measures	Promis-29 instrument, AUDIT-C, PhenX questionnaire	7 domain scores (depression, anxiety, physical function, social function, fatigue, pain interference and sleep); alcohol use disorder screener; tobacco use disorder screener	REDCap
Nutrition Questionnaires	National Cancer Institute ASA24 questionnaire	65 nutrients and 37 food groups	REDCap
Physical activity tracker	PAVS questionnaire; Fitbit activity tracker	Validated physical activity questionnaire; Fitbit online portal	REDCap
Noninvasive liver fibrosis staging	Transient Elastography, Velacur	Liver stiffness measurement Controlled attenuation parameter (CAP) Spleen stiffness measurement Velacur-guided attenuation coefficient estimate (ACE)	REDCap
Cross-sectional abdominal imaging	MRI abdomen with MR elastography and proton density fat fraction	Liver fat fraction Liver stiffness score. Presence of portal hypertension Presence of cirrhosis by morphological evaluation	EHR-PACS
Liver biopsy (when available)	Digital Images	NASH CRN score: steatosis grade (0–3); inflammation (0–3); ballooning (0–2); fibrosis stage (0–4)	EHR
Salivary and Stool samples	16s rRNA metagenomic sequencing	Taxonomic classification and species abundance	Various databases
Plasma, serum	Metabolomics and Lipidomics GC/MS, LC/MS (RP and HILIC)	Metabolite profiles	Various databases
Genotyping	Genotyping chip	Whole-exome sequencing	Various databases
Metabolic and inflammatory proteome	Millipore high-sensitivity multiplex assay	Serum inflammatory and metabolic protein levels	Various databases

Abbreviations: ALT, alanine aminotransferase; ASA24, Automated Self-Administered 24-Hour Dietary Assessment Tool; AST, aspartate aminotransferase; AUDIT-C, Alcohol Use Disorders Identification Test; BMI, body mass index; BUN, blood urea nitrogen; DEXA, dual-energy X-ray absorptiometry scan; ECG, electrocardiography; EHR, electronic health records; GC/MS, Gas chromatography–mass spectrometry; GGT, gamma glutamyl transferase; HDL-C, high-density lipoprotein cholesterol; LC/MS, Liquid chromatography–mass spectrometry; LDL-C, low-density lipoprotein cholesterol; MRI, magnetic resonance imaging; PAVS, physical activity vital sign; NASH CRN, The Clinical Research Network in Nonalcoholic Steatohepatitis; PT/INR, prothrombin time/international normalized ratio; RP/HILIC, reversed-phase liquid chromatography/hydrophilic interaction chromatography.

Before patient-derived PBTs were produced from iPSC-derived liver cells, LAMPS were implemented as a platform for evaluating MASLD progression and drug testing by combining primary human hepatocytes and LSECs and two human cell lines for Kupffer cells and stellate cells. This model has been used to develop the disease model and as a reference for the patient-specific iPSC-derived liver cells used in PBTs. The MASLD-associated genetic variant *PNPLA3* rs738409 (Patatin-like phospholipase domain-containing protein 3) has been investigated as it is clinically associated with more severe MASLD phenotypes and progression to fibrosis and represents an example of genetic heterogeneity found in MASLD patients. 70 71 72 LAMPS were constructed with genotyped wild type (CC) and high-risk GG (I148M) variant PNPLA3 human hepatocytes together with the three nonparenchymal cells. Published MASLD media conditions, 55 67 73 normal fasting (NF), early metabolic syndrome and late metabolic syndrome conditions have been applied to progress the disease in vitro in less than 2 weeks. Levels of steatosis, stellate cell activation and secretion of proinflammatory cytokines and the profibrotic marker COL1A1 in the PNPLA3 high-risk GG variant were shown to be higher compared with those in wild type CC LAMPS, demonstrating a genotype effect on MASLD progression, consistent with clinical observations. 70 74 75 76 77 78 79

**Fig. 4:**
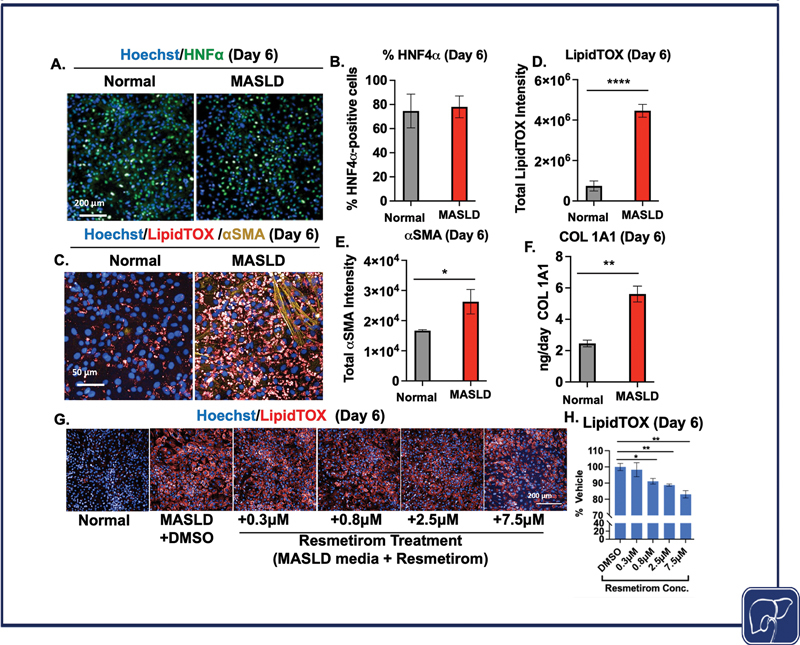
Patient biomimetic twins (PBTs) constructed with patient-specific induced pluripotent stem cell (iPSC)-derived hepatic cells demonstrate increased steatosis under metabolic dysfunction-associated steatotic liver disease (MASLD) medium conditions and show a dose-dependent reduction in steatosis upon treatment with resmetirom. Initial PBTs were constructed in the liver acinus microphysiological system (LAMPS) using iHeps, iStellate, and iEndo-derived from iPSCs and were maintained for 6 days under flow in either normal fasting medium or MASLD medium that was supplemented with increased amounts of glucose, free fatty acids, and TGF-β1. While PBTs display a similar overall percentage of HNF4α iHeps in either media condition, indicating no loss of iHep maturity ( **A; B** ), PBTs maintained in disease media demonstrate a significant increase in steatosis ( **C; D** ; LipidTOX), stellate cell activation ( **C; E** ; αSMA) and collagen 1A1 (COL 1A1) secretion ( **F** ) compared with PBTs maintained in normal medium. This is consistent with our previous work using primary cells using the MASLD LAMPS platform. We also tested the efficacy of a range of resmetirom concentrations (0.3, 0.8, 2.5, and 7.5 μM) to reduce steatosis in PBTs over a 6-day period ( **G; H** ). LipidTOX staining was quantified on day 6 and data were plotted as the average % vehicle ± standard deviation. A two-tailed *t* -test was used to determine significance between control and MASLD medium conditions as well as between vehicle control and resmetirom-treated groups; *p* -values < 0.05 were considered significant. For each group 3 biological replicates were analyzed. Resmetirom treatment resulted in a dose-dependent reduction in steatosis ranging from 10 to 13% (0.8 and 2.5 μM) and a 17% reduction at 7.5 μM. These results are consistent both with our previous studies using resmetirom in primary cell LAMPS where we observed approximately 30% reduction in steatosis with 1-μM resmetirom and with Phase III clinical trial data for resmetirom where a 30% reduction in liver fat was observed in responding patients. 34 73

Resmetirom efficacy was evaluated in the same model as above by comparing responses between the high-risk GG variant and wild-type CC cells treated with 1 µM resmetirom in EMS medium for 8 days ( Supplementary Fig. S2 , available in online version only). Resmetirom increased sex hormone binding globulin secretion in both variants, indicating on-target activity ( Supplementary Fig. S2A , available in online version only). Resmetirom treatment also reduced steatosis ( Supplementary Fig. S2B and C , available in online version only), stellate cell activation ( Supplementary Fig. S2D and E , available in online version only), and COL1A1 secretion ( Supplementary Fig. S2F , available in online version only) to a greater extent in CC LAMPS than in GG LAMPS, consistent with clinical trials. 34 80 81 Interestingly, the change in the cytokine secretion profile in response to resmetirom treatment was more complex, with a greater reduction of IL-8 and CCL-2 in CC genotype while a greater reduction in the secretion of IL-6 was quantified in the GG genotype. These findings demonstrate the capability of the LAMPS platform to identify genotype-specific responses to drug treatment. Success with this cell-based model set up the next step that was to generate a LAMPS with iPSC-derived, patient-specific cells to create PBTs (see Fig. 4 below).

In addition, the effect of liver-derived factors on pancreatic islets in MASLD and T2D comorbidity 62 as well as other diseases have been reported using the coupling of liver and other organ MPS. 82 Importantly, coupling of multiple organ PBTs will be critical to experimentally test the effects of extrahepatic organs ( Fig. 2 ).

## Opportunities and Challenges in the Development and Application of Integrated Patient Digital Twins and Patient Biomimetic Twins as a Precision Medicine Platform for Metabolic Dysfunction-Associated Steatotic Liver Disease

The key goal is to convert the PDT and PBT data into actionable, clinically relevant knowledge through this potentially powerful precision medicine platform to improve the lives of patients and minimize the misdirected use of expensive therapeutics that might not work in an individual patient or specific cohort of patients ( Fig. 1 ).

Conceptually, future databases could include deidentified patient data and PDTs from large cohorts, linked to biobanks of cryopreserved cells (iPSCs/organoids) for generating PBTs. Patient cohort data, PDTs, and PBTs in such databases could be analyzed to identify the most relevant cohort for a new patient profile, potentially reducing the need for extensive, additional PDTs and PBTs while still making patient-relevant predictions. Artificial intelligence could link a new patient dataset to existing PDTs for predictions, with the testing of predictions performed on the closest PBTs using the biobanked iPSCs or specific organ cells. If successful, this approach could significantly reduce time and cost in making predictions and testing predictions.

## Challenges in the Steps Involved in the Development of the Patient Digital Twins–Patient Biomimetic Twins Precision Medicine Platform

There are seven components with unique challenges in the development of the PDT-PBT precision medicine platform: (1) collection of patient data and samples; (2) management of data and samples; (3) production and characterization of iPSC-derived patient cells; (4) generation and application of PBTs; (5) data processing, integration, workflow tools and modeling PDTs; (6) integration of PDTs and PBTs for predicting and testing; (7) ethical, legal, and social implications.

1. Collection of Patient Data and Samples

A key strategy is to begin with a highly engaged and motivated cohort of heterogeneous MASLD patients in the clinic, who will undergo comprehensive clinical phenotyping at baseline before starting MASLD treatment and at prespecified time points throughout the treatment course. This in-depth data collection will allow for a detailed understanding of disease progression and response to treatments. In addition to obtaining the necessary institutional review board approvals, it is important to secure permission for using patient data and samples in collaborative research, particularly with industry partners. This collaboration can help accelerate the testing and refinement of the precision medicine platform, potentially expediting its adoption by the pharmaceutical industry for broader clinical use.

Optimally, deep patient clinomic data are collected that incorporates metrics beyond the minimum for MASLD to have insights into any potential extrahepatic disease, together with metabolomic, lipidomic, and genotype data. In addition, blood samples are collected from each patient for generating iPSCs from isolated peripheral blood mononuclear cells (PBMCs). The deidentified patient data and the deidentified blood samples must be linked for the future development of the PDTs and PBTs.

As an example, a patient cohort population from over 900 enrolled patients in the clinic (UPMC Liver Steatosis and Metabolic Wellness Program) has been selected. 83 84 85 86 87 The initial focus of the enrollment is on patients with MASLD and severe obesity (BMI > 35 kg/m ^2^ ) for whom pharmacotherapy is currently indicated if they have clinically significant liver fibrosis (F2 or higher fibrosis stage). Approximately 84% of patients in this cohort have obesity or are overweight, 45% have Type 2 diabetes, 60% have dyslipidemia, and 55% exhibit hypertension. The cohort is well balanced in terms of age distribution, sex, ethnic, and racial ancestry and presence of metabolic comorbid conditions.

Subjects should undergo detailed morphometric assessments at enrollment (baseline measurements and biospecimen collection), at months 12 (repeat of baseline measurements and biospecimen collection), and month 24. Subjects should also undergo an abbreviated evaluation at month 6, focused on patient-reported outcome measures, evaluation of treatment side effects, noninvasive liver fibrosis and steatosis assessment, and biospecimen collection. This timeline allows comprehensive evaluations on short-term and long-term outcomes that are liver-focused (e.g., steatosis and fibrosis), systemically focused (e.g., body composition and glucose regulation), and patient-reported outcomes (e.g., symptoms, physical activity, and dietary intake) with liver-directed therapies (e.g., thyroid hormone receptor β-agonist, resmetirom) as well as systemically acting drugs (e.g., semaglutide and other incretin agonists).

2. Management of Data and Samples

The management of the data and samples collected from the patients is a significant challenge that includes both ethical and technical issues. Assembling and formatting the disparate data are the critical first step in creating PDTs and requires development of an IT infrastructure to aggregate data from various data sources ( Table 2 ) and harmonize them for computational modeling. 88 A common data model approach should be employed to standardize clinical data from multiple sources. ( Supplementary Fig. S1 , available in online version only). Curation of clinomics data from electronic health record, health survey data, lifestyle data, metabolomics, lipidomics, and genomic data collected from a selected cohort of MASLD patients should be included in a central database ( Supplementary Fig. S1 , available in online version only). In addition, patient reported outcome measures should be available at baseline and at 6-month study visits using the PROMIS-29 instrument, 89 as are patient-reported lifestyle measures from the ASA24 diet questionnaire, 90 physical activity, vital signs, as well as any alcohol, tobacco, or substance use and the Patient Activation Measure and other patient engagement tools. These data should be captured in a comprehensive online federated database such as REDCap. 91 This deeply characterized clinical dataset comprehensively combines a diverse set of metrics, ranging from complex clinical data including clinical notes, laboratory tests, images, medications, clinical outcomes, and lifestyle surveys as well as metabolomic, lipidomic, genomic, cellular and tissue-level omics ( Table 2 ) for the downstream creation of PDTs. Natural language processing and text mining approaches can be employed to extract information from unformatted records such as clinical notes, radiology and pathology reports, and endoscopic procedures to include in the dataset. 92

The biobank resource is a collection of biological specimens, including patient blood, saliva and stool samples, and iPSCs derived from them, along with their deidentified associated data that are used for the creation of PBTs. As with the clinical data, ethics, privacy, informed consent and security need to be addressed concerning the collection and biobanking of clinical samples from patients. 93 94 Standardized procedures and quality control employed when preparing and storing the original clinical samples and patient-derived iPSCs ensure biological samples remain viable for long-term research and provide for reliable and high-quality biological specimens for the generation of future PBTs. The traceability of biological samples and associated deidentified data in the biobank allows for connecting the PBT data with the clinical and PDT data.

Biobanking high-quality cells and their associated data facilitates testing the reproducibility of patient-specific PBTs and the identification of biological heterogeneity across patient PBTs. Biobanking of iPSCs and potentially their differentiated organ cell derivatives involves numerous challenges related to standardization, quality control, and long-term cryopreservation. A major issue is the variability in iPSC lines due to donor-specific genetic and epigenetic backgrounds, which can affect reprogramming efficiency and differentiation potential, complicating efforts to create uniform biobanked resources. 95 Ensuring the authenticity, genetic stability, and pluripotency of stored lines requires rigorous screening and quality assurance protocols, including karyotyping, sterility testing, and pluripotency marker analysis. 96 97 In addition, the biobanking of differentiated cells introduces challenges due to reduced proliferative capacity and higher sensitivity to freezing and thawing, which can affect cell functionality. 98 Furthermore, standardized data collection, harmonized protocols, and ethical frameworks for consent and data sharing are critical to ensure the reproducibility, accessibility, and scalability (e.g., automation) of biobanked iPSC-derived cells.

3. Production and Characterization of Induced Pluripotent Stem Cells C-derived Patient Cells

**Fig. 5:**
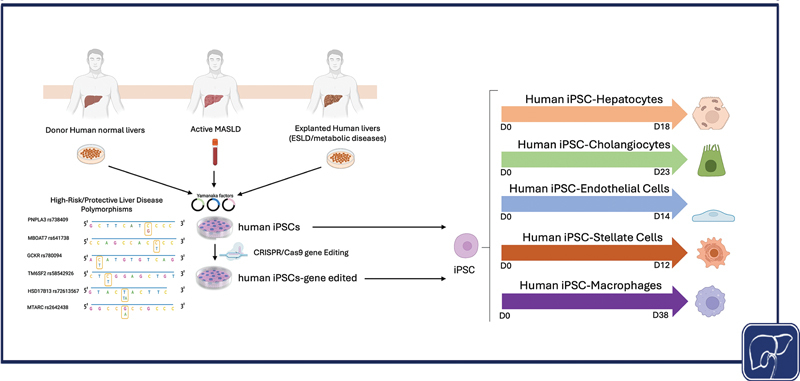
Workflow for the generation of patient induced pluripotent stem cell (iPSC). Blood is collected from each patient in a selected study cohort and peripheral blood mononuclear cells (PBMCs) are isolated ( Fig 1 ). Human-iPSCs are generated from the PBMCs and genotyped. The presence of any known polymorphisms can be corrected using CRISPR/Cas9 gene editing technology. iPSCs are then exposed to a unique set of growth factors, cytokines, and xenobiotics over a multiday period to obtain one of the four hepatic cell types: hepatocytes, liver sinusoidal endothelial cells (LSECs), hepatic stellate cells, and Kupffer cells–macrophages. The final patient biomimetic twin (PBT) is produced through incorporation of iPSC-derived hepatic cell types into a microphysiological system (MPS) in which the disease condition can be driven and quantified and drug testing can be performed.

PBMCs isolated from each enrolled patient's blood serve as starting material for reprogramming into iPSCs (skin fibroblasts can also be used), which are then differentiated into organ-specific cells to create PBTs ( Fig. 5 ). Using validated protocols and standardized operating procedures (SOPs), iPSCs are reprogrammed into a pluripotent state, allowing differentiation into various organ cell types. The present liver cells to be differentiated, include hepatocytes, LSECS, Kupffer cells (macrophages), and stellate cells. Detailed SOPs are required to ensure reproducibility, enabling other investigators to replicate the methods effectively. The goal is to have a set of the most important cells to capture the majority of the clinically relevant functions in the cells. The complexity of the PBTs may increase based on the results with the first-generation system.

During the last several years, human iPSCs have been differentiated into a variety of parenchymal and non-parenchymal liver cells and complex tissues. 99 100 101 102 103 The rationale is that iPSC-derived liver cells from MASLD patients, when exposed to media reflecting the patient disease state, will mimic the functional and phenotypic characteristics of MASLD. 11 66 73 The goal is to have the PBTs created from the patient iPSCs recapitulate what is characterized in mature primary cell MPS experimental models ( Supplementary Fig. S2 , available in online version only). It is a challenge and a requirement to characterize each patient-specific iPSC line for markers of pluripotency. 100 To validate that the iPSC-derived hepatic cells recapitulate the functionality of primary human cells, each cell type must undergo extensive expression and functional analysis prior to PBT construction, including reproducibility metrics (see below). It is important to automate and industrialize the generation of iPSCs and their differentiation into specific organ cells to optimize the reproducibility and scalability to meet the demand of this growing field.

4. Generation and Application of Patient Biomimetic Twins

Historically, the LAMPS and vLAMPS are MPS platforms that have been used to investigate high-content human liver biology and disease using human primary cells and human cell lines. 11 42 63 64 66 69 Both presently contain four primary liver cell types (hepatocytes, LSECS, HSCs, and resident macrophages (Kupffer cells) with the potential to add cholangiocytes and additional immune cells, as needed for specific studies. 11 64 67 ( Supplementary Fig. S2 , available in online version only). Both platforms have distinct advantages, including the relative simplicity of the single-channel LAMPS and the ability to flow media and cells (e.g., immune cells) in the vascular channel that can pass through a filter to reach the hepatic channel of the vLAMPS. 11 42 63 64 A large panel of clinically relevant metrics can be measured in both platforms ( Table 1 ). 11 66 Data are managed in the analytics platform that contains a version of the MPS database, which captures and stores the data and metadata and has tools to analyze and model the data ( Supplementary Fig. S1 , available in online version only). 62 104 105

The well characterized patient, iPSC-derived, differentiated liver cells ( Fig. 5 ) require modification of the normal and disease media and assembly protocols compared with human primary liver cells since they are distinct from primary cells ( Fig. 5 ). We have been evolving the PBTs by optimizing the conditions leading up to the integration of all the cell types. In unpublished data ( Fig. 4 ), we have established that PBTs constructed with 3 iPSC-derived hepatic cells demonstrate increased MASLD phenotypes under MASLD medium conditions (modified-EMS) and show a dose-dependent reduction in steatosis upon treatment with resmetirom. In these initial studies, PBTs were constructed in the LAMPS using iHeps, iStellate, and iEndothelial cells differentiated from iPSCs and were maintained for 6 days under flow in either normal medium or MASLD disease medium that was supplemented with glucose, free fatty acids, and TGF-β1. While PBTs display a similar overall percentage of HNF4α-positive iHeps in either media condition, indicating no loss of iHep maturity ( Fig. 4A, B ; HNF4a), PBTs maintained in disease media demonstrate a significant increase in steatosis ( Fig. 4C, D ; LipidTOX), stellate cell activation ( Fig. 4C, E ; αSMA) and collagen 1A1 (COL 1A1) secretion ( Fig. 4F ) compared with PBTs maintained in control medium (modified-NF), consistent with key clinical MASLD phenotypes, as well as our previous work using primary cells using the MASLD LAMPS platform ( Supplementary Fig. S2 , available in online version only). 66 73

We also evaluated the efficacy of resmetirom recently approved for the treatment of MASH 33 in PBTs that were maintained in disease medium for 6 days ( Fig. 4G, H ). PBTs were maintained for 6 days in disease medium containing a dose range of resmetirom (0.3, 0.8, 2.5, and 7.5 μM) or dimethyl sulfoxide vehicle control. LipidTOX staining was performed on day 6 and data were plotted as the average % vehicle ± standard deviation. Resmetirom treatment resulted in a dose-dependent reduction in steatosis ranging from 10 to 13% (0.8 and 2.5μM) and a 17% reduction at 7.5 μM. These results are consistent both with our previous studies using resmetirom in primary cell LAMPS where we observed approximately 30% reduction in steatosis with 1μM resmetirom ( Supplementary Fig. S2 , available in online version only) 73 and with Phase III clinical trial data for resmetirom where a 30% reduction in liver fat was observed in responding patients. 34 81 Therefore, the initial PBTs show great promise in being able to recapitulate patient disease and to respond to therapeutic treatments similar to primary cells ( Supplementary Fig. S2 [available in online version only] and Fig. 4 ). The next version of the PBTs must continue to evolve to include the most physiologically relevant cell types. An important challenge is demonstrating that the compressed time period of disease progression in PBTs maintains the disease state of the patient based on media contents and other variables. This requires that the mechanisms underpinning the pathophysiology in the clinic are preserved in the PBTs through functional and genomic analyses of the cells.

Another major challenge for fully harnessing the PBTs for precision medicine is to demonstrate an acceptable reproducibility so that biologically and clinically relevant heterogeneity between patient-derived PBTs and specific treatments can be quantified. 11 106 PBT studies are “experiments” where the patient-specific PBTs are subjected to different treatments (e.g., cell types included, media conditions, drugs) and typically involve several replicates within different treatment groups. Intrastudy reproducibility refers to running replicate samples within a treatment group within a study and obtaining results that are in close agreement. Interstudy reproducibility refers to performing a study under identical conditions multiple times on different days and in different laboratories and obtaining consistent results across the studies. Several factors contribute to the interstudy experimental reproducibility or lack thereof, including variable cells, reagents, assays, and experimental and analytical methods. Biological heterogeneity is a fundamental property of biological systems manifested at the omics level (e.g., clinomic, genomic, metabolomic, lipidomic) and expressed at the cellular, tissue, organ, and system levels leading to functional/phenotypic variations in normal and disease states. 106 Clinical heterogeneity stems from the biological heterogeneity and manifests as differences in patient characteristics, including presence of clinically relevant metrics of any comorbidities, rate of disease progression, stage of disease, and responsiveness to therapeutic interventions. 107 108 Distinguishing experimental variability from biological/clinical heterogeneity is key for the implementation of precision medicine to address clinical heterogeneity. Quantitation of PBT reproducibility facilitates the validation and qualification of experimental models for ADME/TOX and efficacy in drug discovery and development, helps to inform the optimized enrollment of patients in clinical trials, and assists in selecting optimal therapeutic strategies for distinct patient cohorts. To this end, automation and industrialization of the steps involved in the assembly, processing and analyzing multiple PBTs will be required.

Assessing PBT reproducibility requires knowing details about how the PBT model is configured (metadata) including numbers of each cell type used and how they are incorporated into the model, what experimental parameter settings (e.g., media conditions, flow rate, temperature, cell passage number) are applied, and how the metrics were quantified. For all studies using PBTs it is critical to capture detailed metadata (1) how the PBTs are constructed; (2) the cell samples used in the PBTs including clinomic, genotype, metabolomics, and lipidomics information; (3) experimental metadata describing how the study was performed; and (4) what assays were run and how they were analyzed. We have developed a standard approach called the Pittsburgh Reproducibility Protocol that uses a set of common statistical metrics, the coefficient of variation, analysis of variance, and intraclass correlation coefficient, in a novel workflow to evaluate the intra- and interstudy reproducibility of any MPS or PBT performance. 109 A key challenge will be to demonstrate the reproducibility of the steps from producing iPSC cell lines, differentiating different iPSC lines into organ-specific cells, functions of the differentiated cells alone and assembled into PBTs. In addition, attaining an acceptable reproducibility of coupled organ PBTs to explore extrahepatic disease will be more challenging than the single liver PBTs given the added complexity.

5. Data Processing, Integration, Workflow Tools and Modeling PDTs

In a recent study, 110 it was demonstrated in a patient cohort exhibiting obesity that MASLD encompasses at least two distinct subtypes with similar liver phenotypes at baseline but each with specific liver transcriptomic and plasma metabolic profiles and different clinical trajectories. 110 The first subtype, termed liver-specific, was genetically linked and showed rapid progression of chronic liver disease but limited risk of cardiovascular disease. 110 The second, cardiometabolic, was primarily associated with dysglycemia and high levels of triglycerides, leading to a similar incidence of chronic liver disease, but a higher risk of cardiovascular disease and T2D. 110 This study suggested the need for subtype-selective therapeutic strategies with the authors hypothesizing that resmetirom might be more efficacious for patients with the liver-specific subtype, whereas in contrast, FGF21 analogs, pan-PPAR agonists, and drugs associated with weight loss might be more efficacious in those patients with the cardiometabolic subtype. The unsupervised clustering performed in this study to identify distinct MASLD subtypes used six simple clinical variables: age, BMI, HbA1c, alanine aminotransferase, low-density lipoprotein, and circulating triglycerides. We anticipate that constructing PDTs using additional comprehensive datasets that include more clinomic profiles, as well as transcriptomic, metabolic, and lipidomic profiles will delineate additional MASLD subtypes and generate causal inferences for these subtypes that can then be experimentally tested in corresponding PBTs.

There are multiple technical requirements to optimally harness varied patient data. An application programming interface is required to export clinical data from the analytics platform ( Supplementary Fig. S1 , available in online version only) to local computers for the generation of the PDTs. Clinomics need to be preprocessed into a tabular format consisting of continuous, ordinal, and categorical variables. All categorical variables also need to be converted into binary dummy variables to enable downstream processing by machine learning algorithms. All clinomics, genomics, metabolomics, and lipdomics datasets should be imported into a selected programming language (e.g., R programming language) for analyses. All large-scale biological omics datasets that cannot fit into remote access memory need to be read directly from disk using tools such as the bigstatsr package. Custom statistical algorithms that learn optimal outcome measures can be produced using a variety of programming languages (e.g., R programming language) and thus also perform digital twin simulations using the same programming language. Statistical hypothesis testing using exact permutation methods that account for machine learning model selection need to be applied.

6. Integration of Patient Digital and Biomimetic Twins for Predicting and Testing

An example of applying the integrated PDTs and PBTs to an important clinical challenge is based on the fact that drugs currently under development for MASLD and related metabolic-linked diseases are expected to benefit only a subset of patients due to the factors involved in patient heterogeneity, and it is not possible to predict response a priori. The ability to develop predictive models (PDTs) of therapeutic responses to newly approved drugs and those in the pipeline and then testing them in PBTs, both in liver alone and coupled organ PBTs involved in the CKM syndrome, will be transformative. Individual patients would then be given therapeutics/combinations with the highest probability of being efficacious with no serious safety concerns. This will benefit the patients, physicians, and payers. Although not straightforward, pharmaceutical companies could benefit from developing multiple therapeutics/combinations for the CKM syndrome, a huge global market. Successful application of the integrated PDTs and PBTs could also lead to defining a small set of biomarkers that could be used in stratifying patient cohorts.

It is important to accurately predict the current and future status of the patient's liver with PDTs. To achieve this, clinomic, genomic, lipidomic, and metabolomic data are integrated into a unified tabular dataset, consisting of both discrete and continuous variables, with potential missing values. Given these complexities, PDTs should be constructed using nonlinear machine learning algorithms known for their effectiveness on such datasets. 111 112 It is anticipated that the deep clinomics together with the genotype, metabolomics and lipidomics will create valuable PDTs that reflect a significant aspect of the patient. However, additional patient omics data and time points may be required to optimize the PDTs. This is a significant challenge given the time and financial cost of generating the patient data.

Another challenge will be based on the anticipated highly non-linear models that will require the quantification of each variable's contribution to the prediction task. Tree-based algorithms, including XGBoost 113 and Random Forest 114 should be used to achieve the goals. Tree-based algorithms construct multiple decision trees, which can naturally accommodate missing values, mixed data types and the high dimensionality commonly seen in biomedical data 114 ( Fig. 3A ). The algorithms also exhibit competitive performance across a variety of different tasks on tabular datasets. 112 Finally, tree-based models enable fast estimation of variable importance using the SHapley Additive exPlanations (SHAP) variable importance measure. 115 Importantly, the SHAP measures take on a value that is larger in magnitude when changing the value of the variable creates large changes in the output of the model. 116 Hence, multiple decision tree models achieve the necessary robustness, accuracy, and transparency.

PDTs are used to perform causal inference by estimating patient-specific treatment responses. Importantly, many existing and potential future therapies do not just target liver metrics (e.g., fibrosis) but can impact many different components of the CKM syndrome. 117 Deep phenotypes of the patients are collected going beyond liver-focused MASLD to include metrics involved in various comorbidities ( Table 2 ). In addition, the comorbidities are expected to present different metabolic/lipidomic profiles. PDTs that recapitulate treatment response must therefore adequately model some key components of the CKM syndrome. An optimal approach is to model the components using the SV algorithm, 52 which adopts the model shown in Fig. 3B . The primary and secondary outcome measures encapsulate key components of the metabolic syndrome, such as liver stiffness, hepatic fat fraction, weight, skeletal muscle mass, and glucose levels. Unfortunately, naively using all these outcome measures limits our ability to detect significant treatment effects due to the loss of statistical power with multiple comparisons. 118 As a result, SV instead compresses the many original outcome measures into a small number of optimal outcomes (red in Fig. 3B ) that maximally differentiate between the treatments (green). The algorithm ensures detection of causal treatment effects by adjusting for confounders (purple). SV finally estimates personalized causal treatment effects for the PDTs by finding moderators—or variables that can modify the effect of treatment, 14 such as genetic variants or nascent lipid levels—in the genomic, lipidomic, and metabolomic data.

The data from the PBTs ( Table 1 ) should also be used to generate PBT-based PDTs to determine how well the initial PBT experimental models recapitulate the patient that they are designed to model. This is a critical challenge that defines how well the initial PBTs recapitulate the patient. Limited overlap between the PBTs and the PDTs will require the further optimization of the PBTs in terms of variables, including cell types added to the liver PBT, media, matrix, and the coupling of other organ PBTs (e.g., pancreatic islets, kidney, heart). 62 82 119 120 The tools are available, but the goal is to create a powerful precision medicine platform with the smallest number of components.

The statistical significance of personalized causal effects can be assessed via two-sided permutation testing ( Fig. 3C ). Permutation testing ensures accurate *p* -value estimation under minimal assumptions. 121 The null hypothesis of treatment exchangeability is adopted and, therefore, always permute treatment assignment. The regression coefficient of the treatment–moderator interaction term *T × M* , where *T* denotes binarized treatment and *M* the moderator. Many treatment–moderator interactions must be tested and thus correct for multiple comparisons using the false discovery rate. 122 Moderators that achieve a false discovery rate below the classical 0.05 threshold are then tested in PBTs for experimental verification. If PBTs fail to recapitulate the PDT moderators in vitro, then the moderator is labeled as a potential confounder and the analysis must be repeated. As a result, even negative experimental results from the PBTs improve the PDT model.

Another challenge is that the predictive function of PDTs and the testing with PBTs carries the risk of being biased toward individuals and populations based on the data used in training the models. The example study outlined above involves a statistically valid population of patients based on a focused patient profile. However, the potential power of the precision medicine platform should be greater with a broader population of patients, requiring more enrolled patients.

7) Ethical, Legal, and Social Implications

Ethical and legal considerations are essential when using patient data and samples. The development of PDTs and PBTs is associated with several ethical, legal, and social implications, both positive and negative. Digital twins in the healthcare setting have several socioethical benefits for patient health, potential cost reduction of therapeutic development and delivery, patient autonomy, as well as for fair and equal treatment of patients with minimized animal testing. 123 The socioethical benefits of PDTs and PBTs are based on creating a precision medicine platform that facilitates more efficient and precision drug discovery and development by better understanding the heterogeneity of disease mechanisms, improving clinical trial designs through better cohort selection, identify efficacious therapeutic strategies for individual patients or cohorts, reduce unnecessary cost and suffering to patients by avoiding inefficacious treatments, and reduce the involvement of animals in the drug discovery and development process.

However, PDTs and PBTs also come with several socioethical risks, including those centered around privacy and security, data quality, and inequality and injustice. 123 124 Privacy issues from the patient perspective include knowing what data will be collected, where the data resides, who can access them, how they will be used, who owns the primary data and subsequent data generated from the primary data, and who will benefit from their use. These issues need to be addressed with the patients when obtaining informed consent to release their deidentified data for use in development of PDTs and PBTs, sharing the data and results with the academic community and potentially with industry partners that can accelerate the translation of the results into solutions for patients. Data use agreements also need to be written to handle the internal and external research collaborators use and access to this sensitive and valuable PDT and PBT data, as well as computational models and predictions. Data “hypercollection” collecting data not relevant to the project objectives, is another issue for privacy and patient autonomy. We review all available data and establish what is necessary for building the PDTs and PBTs and eliminate hypercollection. It is valuable to work closely with the Research Protection Office at the institution where the study is centered and also include patients, caregivers, and patient advocacy groups working closely with the clinical research team to provide feedback on the study design, data collection, and safety and comfort of patients.

Security is another major concern for health-related data. The data security strategy should use a “Defense in Depth” strategy that puts in place a series of security controls. A threat that manages to circumvent one control is likely to be thwarted by a control in another layer. The Defense in Depth strategy relies on multiple defensive mechanisms, at multiple layers, performing different tasks. Network firewalls are the first line of defense and provide the greatest level of protection from attacks originating on the Internet. The IT team must proactively detect and remediate attacks on the network.

Finally, low-quality data can create PDTs that poorly represent their physical counterpart and lead to erroneous predictions. Clinical and research data from diverse sources often lacks standardization, and it is difficult to assess the reliability and cleanliness of the data. Harmonization and standardization of the data are required to provide quality data for PDTs. 8 9 Therefore, a data governance program must be established to manage the handling of the clinical and research data, ensure maintenance of their integrity from the data source to the Pitt analytics platform.

## Concluding Remarks

There is great potential for combining and integrating PDTs and PBTs as a powerful precision medicine platform to optimize patient-specific drug discovery and development, as well as patient-specific therapeutic strategies, especially for complex, heterogeneous diseases. The potential to decipher distinct mechanisms enables the identification of optimal targets for an individual patient, along with companion biomarkers directly linked to the cause, in contrast to the effect of the pathophysiology. This helps to ensure that the pharmacodynamic effect of the therapy will be directly connected to the root cause of the disease resulting in predictable disease modification in contrast to just the alleviation of clinical phenotypes. In addition, the stratification of patients based on disease mechanism and drug mode of action will lead to more optimal clinical trial designs and therapeutic strategies for individual patients.

However, success will require solving a variety of technical and ethical challenges. This perspective focused on MASLD as an example, but any complex, heterogeneous disease would benefit from this approach, including all cancers, cardiovascular diseases, some autoimmune diseases, and psychiatric diseases. Deep clinomics data together with relevant omics data are required to generate strong predictive PDTs and single or multiple organ PBTs that recapitulate the disease must be validated and exhibit reproducibility.

## Notes

Lay Summary The present methods to discover and develop new therapeutics have been very costly, time-consuming, and inefficient. This is especially true for complex diseases where patients have genetic variations of key disease-related genes, different lifestyles including eating and drinking habits, different environments, such as what bacteria inhabit their intestines, as well as having multiple, related diseases, such as fatty liver disease and type 2 diabetes (T2D). Recently, there has been a shift from the sole reliance on animal models of disease and toxicology to new approach methods (NAMs) that include computational models of patients based on clinical data and other patient-specific data, called patient digital twins (PDTs), that are being developed to make predictions about the patients. In addition, the use of cells from patients to construct experimental models called patient biomimetic twins (PBTs) that reflect the biology and illness of individual patients can be used to test the predictions on each patient. The integration of PDTs and PBTs has the potential to create a powerful precision medicine platform.
