# Supplementary material for: Integrated Patient Digital and Biomimetic Twins for Precision Medicine: A Perspective
Source: Semin Liver Dis. 2025 Jul 23;45(4):458–75. doi: 10.1055/a-2649-1560 (PMC12312794; doi:10.1055/a-2649-1560)
Supplement: Supplementary file 1 — Supplementary Material [file 10-1055-a-2649-1560-s2500036.pdf]

Thieme

Supplementary Material

Integrated Patient Digital and Biomimetic Twins for Precision Medicine: A Perspective

Mark T. Miedel, PhD, Mark E. Schurdak, PhD, Andrew M. Stern, PhD, Alejandro Soto-Gutierrez, MD, PhD*, Eric von Strobl, MD, PhD*, Jaideep Behari, MD, PhD*, D. Lansing Taylor, PhD*

Corresponding Author:
D. Lansing Taylor, PhD, Distinguished Professor and Allegheny Foundation Professor of Computational and Systems Biology, Organ Pathobiology and Therapeutics Institute, University of Pittsburgh, 4107 Pittsburgh Technology Center, 700 Technology Drive, Pittsburgh, PA 15219
dltaylor@pitt.edu
412-916-3129

Co-Authors:
Mark T. Miedel, PhD, Assistant Professor, Department of Pharmacology and Chemical Biology, Organ Pathobiology and Therapeutics Institute, University of Pittsburgh, Pittsburgh, PA

Mark E. Schurdak, PhD, Associate Professor, Department of Computational and Systems Biology, Organ Pathobiology and Therapeutics Institute, University of Pittsburgh, Pittsburgh, PA

Andrew M. Stern, PhD, Associate Professor, Department of Computational and Systems Biology, Organ Pathobiology and Therapeutics Institute, University of Pittsburgh, Pittsburgh, PA

Alejandro Soto-Gutierrez, MD, PhD*, Professor of Pathology, Endowed Chair of Experimental Pathology. Department of Pathology, Center for Transcriptional Medicine, University of Pittsburgh, Pittsburgh, PA

Eric von Strobl, MD, PhD*, Assistant Professor, Department of Biomedical Informatics, University of Pittsburgh, Pittsburgh, PA

Jaideep Behari, MD, PhD*, Professor of Medicine, Department of Medicine, Division of Gastroenterology, Hepatology and Nutrition, University of Pittsburgh, Pittsburgh, PA

* Co-Senior Authors

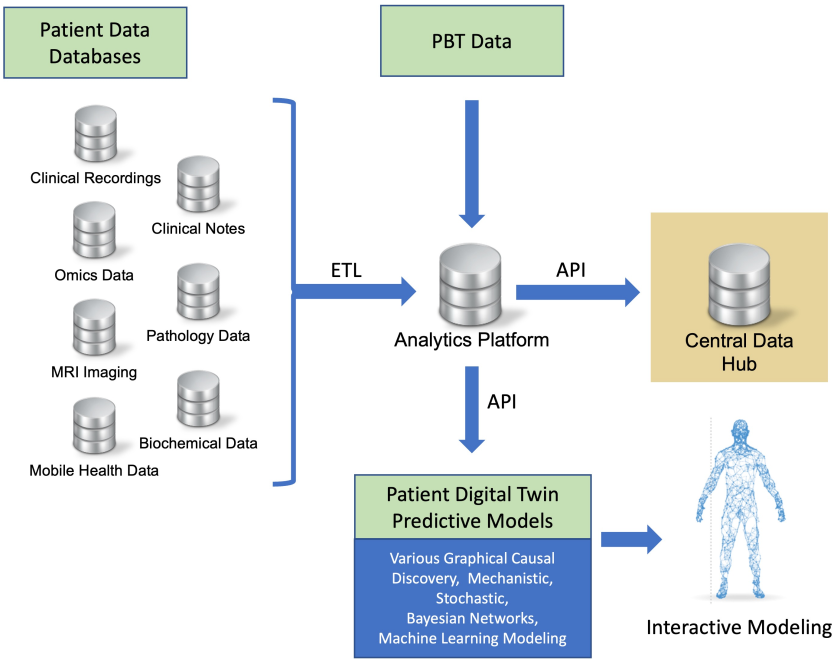

Figure S1. Overall design for integration of clinical and PBT data for the creation of PDT and data dissemination. Multimodal MASLD patient data residing in multiple clinical databases will be brought together in an analytics platform utilizing ETL processes to preprocess the data including cleaning and standardizing terminologies, ontologies, and formatting. Through the creation of Application Programming Interfaces (APIs), the clinical data will be made available for downstream computational modeling and creation of the PDTs, which will enable the prediction of diagnosis, prognosis, treatment strategies, drug response, and clinical biomarkers, as well as selecting cohorts for clinical trials. In vitro experimental data from PBTs will also be entered into the analytics platform, which includes a version of the MPS database or a similar database, for analysis and dissemination. All data will be part of a Central Data Hub.

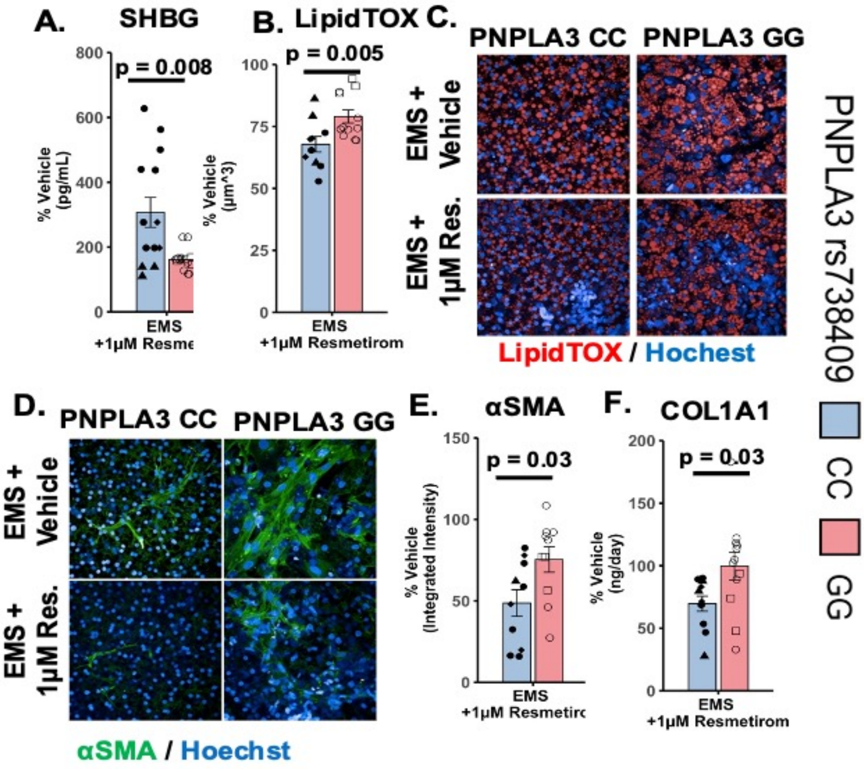

Figure S2. Resmetirom treatment resulted in a greater reduction of steatosis, stellate cell activation and COL1A1 secretion in PNPLA3 CC wild type compared to GG variant in primary cell LAMPS. (A) 1 µM resmetirom treatment increased SHBG secretion in both PNPLA3-LAMPS **(B)** Resmetirom treatment resulted in a significantly greater reduction in steatosis in PNPLA3 CC wild type compared to PNPLA3 GG variant. **(C-D)** Representative images of LipidTOX and αSMA labeled PNPLA3-LAMPS maintained in EMS with and without 1µM resmetirom. **(E-F)** A significantly greater reduction in both αSMA integrated intensity and the secretion of COL1A1 was observed in PNPLA3 CC wild type compared to PNPLA3 GG variant LAMPS. See the discussion of the impact on cytokines in the section titled: Background to the Development of Patient Digital and Biomimetic Twins. This figure has been adapted with permission from our previously published work[1], (Xia M, *et al*. Front Cell Dev Biol. 2024 Sep 11;12:1423936. PMID: 39324073)

Supplementary References

1. Xia M, Varmazyad M, Pla-Palacin I, et al. Comparison of wild-type and high-risk PNPLA3 variants in a human biomimetic liver microphysiology system for metabolic dysfunction-associated steatotic liver disease precision therapy. *Front Cell Dev Biol*. 2024;12:1423936. doi:10.3389/fcell.2024.1423936
